# Traumatic Retroclival Subdural Hematoma

**DOI:** 10.5334/jbsr.3163

**Published:** 2023-08-30

**Authors:** Tom Van der Stricht, Floris De Munck, Koenraad Nieboer

**Affiliations:** 1 Universitair Ziekenhuis Brussel, Belgium

**Keywords:** Retroclival hematoma, subdural hematoma, trauma, CT, MRI

## Abstract

**Teaching Point:** Retroclival subdural hematoma is a rare type of extra-axial hematoma after craniocervical trauma.

## Case History

A 73-year-old woman was admitted to the emergency department after a fall down the stairs in which she suffered a head injury. A deep skin laceration was visible on the forehead.

She complained about a mild headache and neck pain. However, she did not lose consciousness, and there were no signs of neurological deficit.

Several years ago, she had a history of anterior cervical discectomy and fusion at level C5-C6.

**Figure 1:**
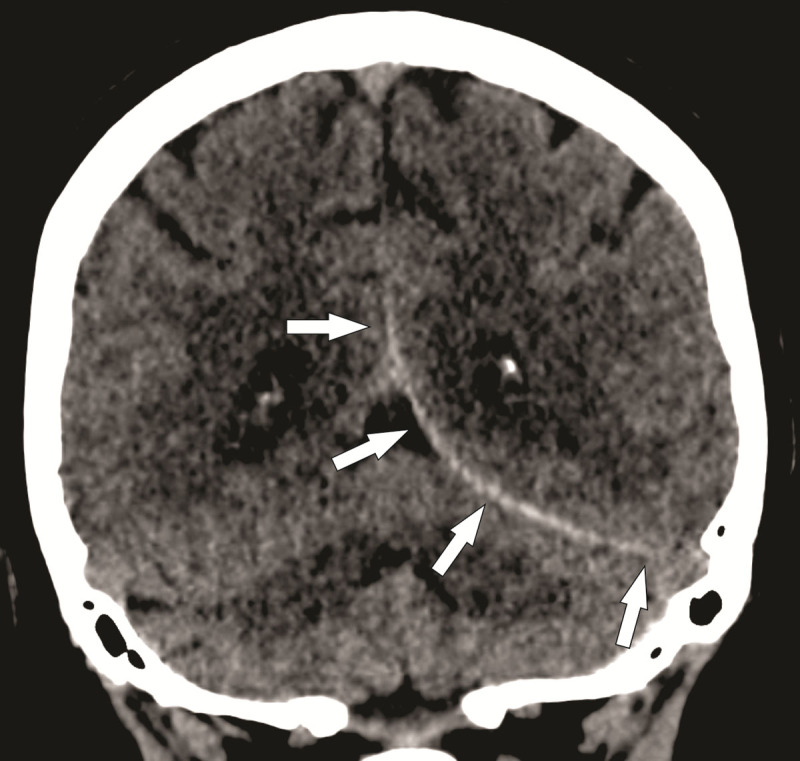


Computed tomography (CT) revealed no skull or cervical spine fractures, but a small subdural hematoma was seen along the left tentorium without significant mass effect (Figure 1, white arrow).

**Figure 2:**
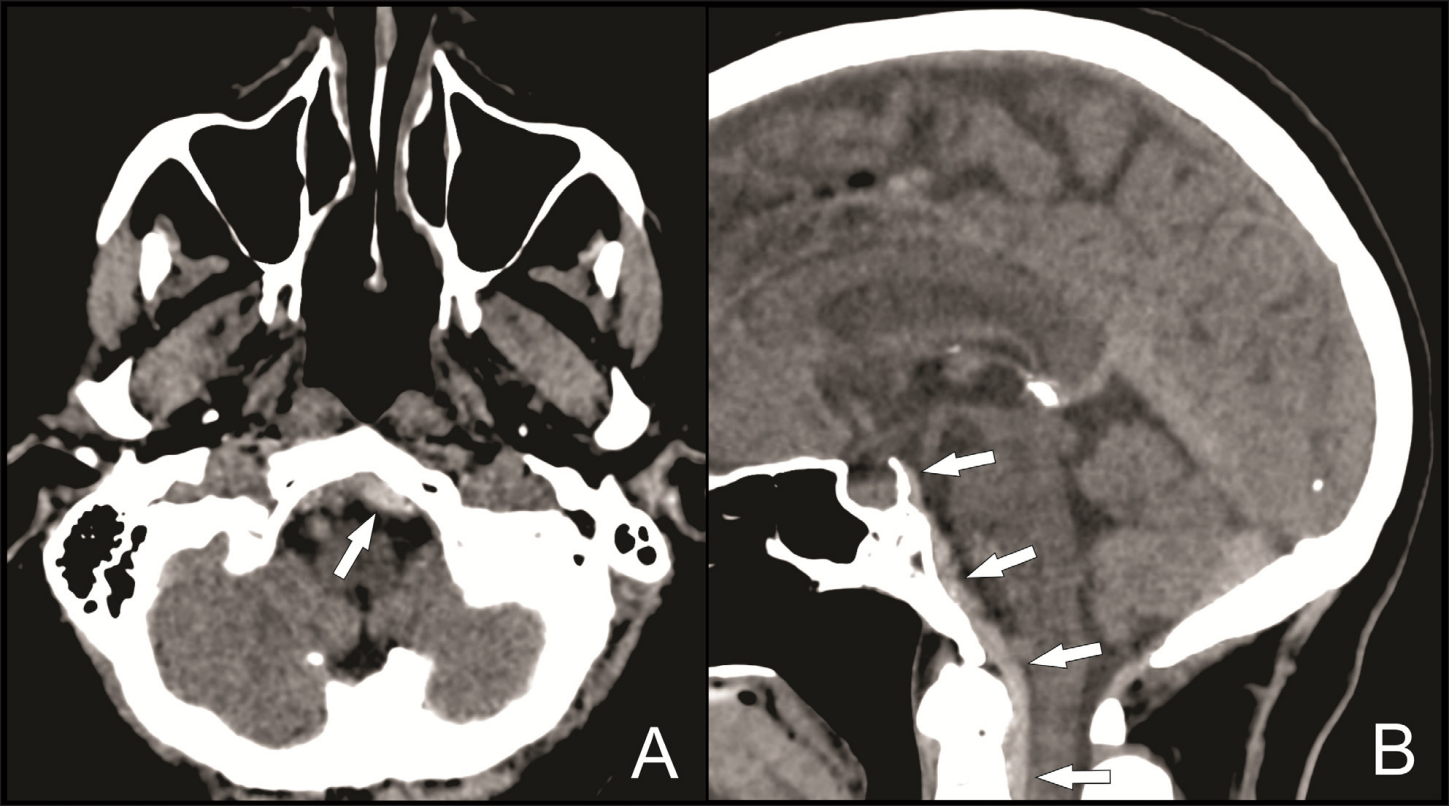


In addition, a hyperdense collection with a maximal thickness of 6 mm was visible posterior to the clivus and descending anteriorly into the foramen magnum (Figure 2A–2B, white arrows), suggestive of an acute subdural or epidural hematoma.

**Figure 3:**
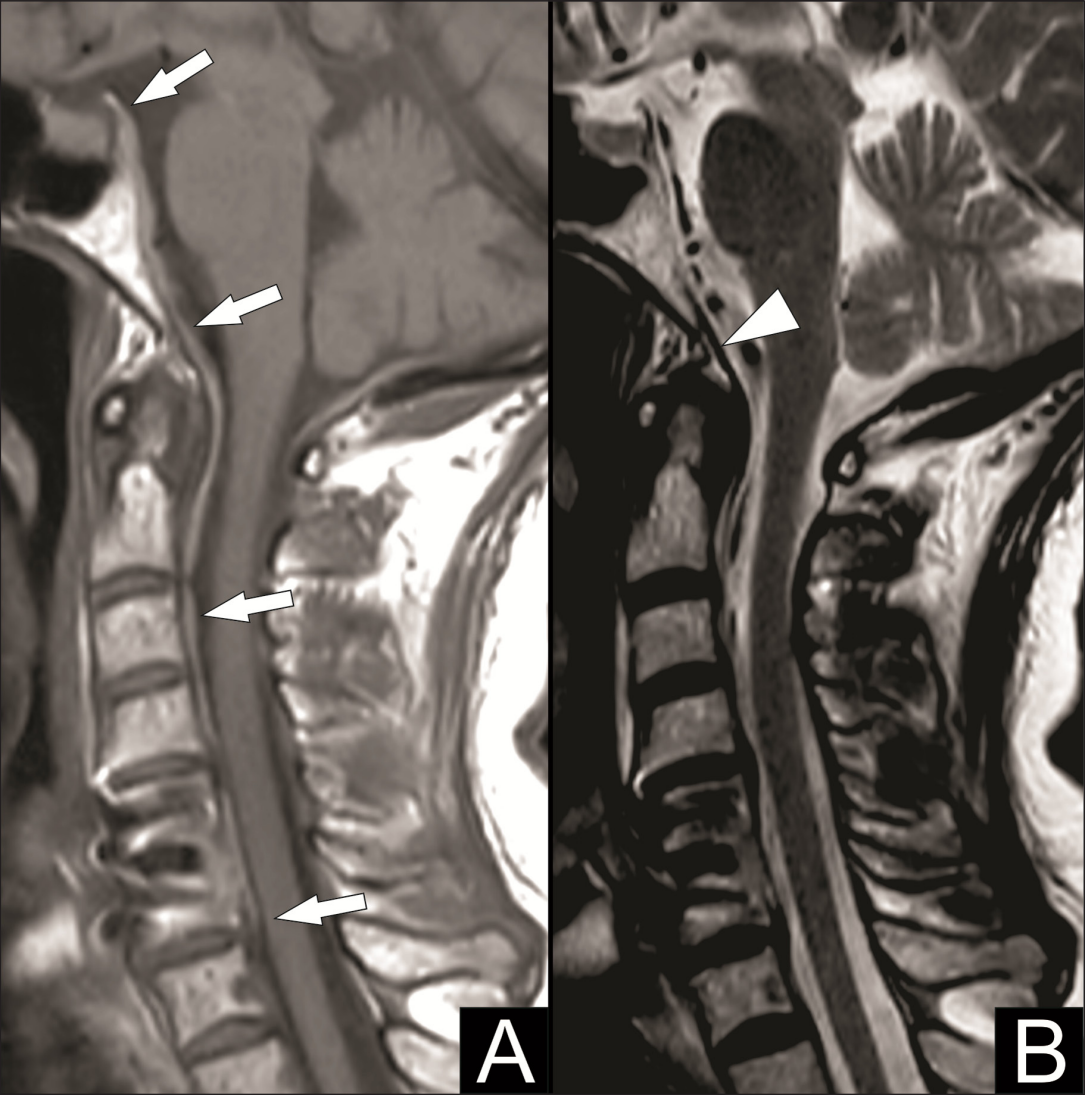


Magnetic resonance imaging (MRI) of the cervical spine was then performed to assess this collection better. MRI showed a hematoma posterior to the tectorial membrane descending anteriorly into the spinal canal up to the posterior body of C6, best seen on T1-weighted images (Figure 3A, white arrows), and an intact tectorial membrane, best seen on T2-weighted images (Figure 3B, white arrowhead). These imaging findings are suggestive of a retroclival subdural hematoma.

The patient was hospitalized for observation but could leave the hospital in good condition after a few days without surgical intervention.

## Comments

Retroclival hematomas are rare and represent less than 0.3% of acute extra-axial hematomas. Adult retroclival hematomas are even rarer. Most case reports in the literature involve pediatric patients. This is due to different craniocervical anatomy in children. We can categorize retroclival hematomas into subdural and epidural hematomas, with the latter being more frequent. The most common cause is craniocervical trauma. Other possible causes include pituitary hypoplexia and coagulopathy [1].

The tectorial membrane is vital in distinguishing between a subdural and an epidural retroclival hematoma. The tectorial membrane extends from the posterior longitudinal ligament and attaches to the posterior body of C2 and the clivus. Because of a tight adherence of the spinal dura to the tectorial membrane at the C2 level, epidural retroclival hematomas cannot descend below this C2 level. Subdural hematomas, on the other hand, are not restricted by the tectorial membrane and can descend below the C2 level into the spinal subdural space. Furthermore, the tectorial membrane is often ruptured with an epidural hematoma and remains intact with a subdural hematoma [1].

Diagnosis can be made with CT, but MRI is often performed to assess for ligamentous lesions and possible compression on the cranial nerves and brainstem. Sagittal reconstructions are essential to recognize a retroclival hematoma. Most patients are treated conservatively and have good clinical outcomes.
